# Marker-assisted breeding of Indonesia local rice variety Siputeh for semi-dwarf phonetype, good grain quality and disease resistance to bacterial blight

**DOI:** 10.1186/s12284-014-0033-2

**Published:** 2014-12-18

**Authors:** Yanchang Luo, Sabaruddin Zakaria, Bakhtiar Basyah, Tingchen Ma, Zefu Li, Jianbo Yang, Zhongchao Yin

**Affiliations:** Temasek Life Sciences Laboratory, 1 Research Link, National University of Singapore, Singapore 117604 Republic of Singapore; Department of Agrotechnology, Agriculture Faculty, Syiah Kuala University, Darussalam-Banda, 23111 Aceh, Indonesia; Rice Research institute, Anhui Academy of Agricultrural Sciences, Hefei, 230031 China; Department of Biological Sciences, 14 Science Drive, National University of Singapore, Singapore 117543 Republic of Singapore

**Keywords:** Rice, Marker-assisted selection, Semi-dwarf, Amylose content, Bacterial blight

## Abstract

**Background:**

Rice is one of the most important staple food crops in Asia. Since the first green revolution beginning in 1960s, high-yield semidwarf modern rice varieties have been widely planted; however, traditional rice varieties with tall plant type are still grown in many countries due to their good grain quality and adaptation to local climate and environment. Siputeh, a local rice variety mainly planted in Java and Sumatra islands of Indonesia, produces long grain rice with good cooking and eating quality. However, the variety has low yield with tall plant type and long growth duration and is highly susceptible to biotic and abiotic stress.

**Results:**

Siputeh as the recurrent female was crossed with the donor line WH421, an elite paternal line of hybrid rice containing the *sd1*, *Wx*^b^, *Xa4* and *Xa21* genes, followed by backcrossing and self-pollination. TS4, a BC3F4 line derived from the breeding program, was obtained through marker-assisted selection for the *sd1*, *Wx*^b^, *Xa4* and *Xa21* loci. TS4 has semi-dwarf phenotype and short growth duration. TS4 conferred disease resistance to multiple *Xanthomonas oryzae* pv. *oryzae* (*Xoo*) strains collected from different countries around the world. TS4 achieved higher grain yield than Siputeh in two field trials conducted in Banda Aceh, Indonesia and Lingshui, China, respectively. Finally, TS4 has better grain quality than Siputeh in terms of degree of chalkiness and amylose content.

**Conclusion:**

An improved rice line, designed as TS4, has been developed to contain semi-dwarf gene *sd1*, low amylase content gene *Wx*^b^ and bacterial light resistance genes *Xa4* and *Xa21* through marker-assisted selection. TS4 has semi-dwarf phenotype with reduced growth duration, produces high yield with good grain quality and provides broad-spectrum resistance to *Xoo* strains. The development of TS4 enriches the diversity of local rice varieties with high yield potential and good grain quality.

**Electronic supplementary material:**

The online version of this article (doi:10.1186/s12284-014-0033-2) contains supplementary material, which is available to authorized users.

## Background

Rice (*Oryza sativa* L.) is a staple food crop in most of the Asia countries. The introduction of semi-dwarf rice led to record yield increases throughout Asia in the 1960s. The recessive semi-dwarfing gene, *sd-1*, is one of the most important genes deployed in modern rice breeding. Rice varieties with the *sd1* gene have a shortened culm with improved lodging resistance and a greater harvest index, allowing for the increased use of nitrogen fertilizers (Jennings [1964]). The *sd1* gene has been cloned from rice and its dominant wild-type allele encodes a gibberellin (GA)-20 oxidase-2 (GA20ox-2) (Ashikari et al. [2002]; Sasaki et al. [2002]; Spielmeyer et al. [2002]). The deletion or substitution mutation of the *Os20ox2* gene results in loss of function of the enzyme, which ultimately leads to a deficiency in plant growth hormones gibberellins that control plant height (Ashikari et al. [2002]; Sasaki et al. [2002]; Spielmeyer et al. [2002]). The different alleles of the *sd1* gene have remained the predominant semi-dwarf genes in modern rice cultivars (Asano et al. [2007]).

There are two different types of starch, amylose and amylopectin, in rice endosperm and the amylose content affects rice eating and cooking quality (Cruz and Khush [2000]). With content ranging from 5% to 15%, the low-amylose rice is characterized by fluffy texture, glossy appearance of the cooked rice, soft texture of cooled rice, and excellent expansibility for food processing (Zhu et al. [2003]). Genetic studies have shown that the rice *Wx* gene determines the amylose content in the endosperm. *Wx* encodes granule-bound starch synthase I (GBSSI), a key enzyme for amylose biosynthesis in the endosperm, and *wx* mutant endosperm contains almost exclusively amylopectin (Hori et al. [2007]; Sano [1984]; Wang et al. [1995]). In addition to controlling amylose content, *Wx* also affects gel consistency and gelatinization temperature of rice starch (Su et al. [2011]). *Wx*^a^ and *Wx*^b^ are two predominantly distributed functional *Wx* alleles in rice (Sano [1984]; Wang et al. [1995]). *Wx*^a^ is widely distributed in *indica* rice with high amylose content while *Wx*^b^ is mainly found in *japonica* rice with low or intermediate amylose content (Sano [1984]; Wang et al. [1995]). A single nucleotide substitution (G-to-T) at the splice donor site of the first intron in *Wx*^b^ potentially alters the splicing site and decrease the splicing efficiency of the first intron of *Wx*, resulting in the low level of both mature transcript of *Wx*^b^ and amylose content (Cai et al. [1998]; Larkin and Park [1999]; Tian et al. [2009]; Wang et al. [1995]). Based on the polymorphism at the splicing site of the first intron, a co-dominant CAPS (cleaved amplified polymorphic sequence) molecular marker PCR-*Acc* I was developed for selection of *Wx* alleles in rice breeding (Cai et al. [2002]).

Bacterial blight of rice, caused by *Xanthomonas oryzae* pv. *oryzae* (*Xoo*), is one of the most important bacterial diseases prevalent throughout the world (Gnanamanickam et al. [1999]). Rice yield losses caused by bacterial blight can reduce yield by 20-30% and by up to 50% in some areas of Asia (Mew et al. [1993]). The utilization of host disease resistance (*R*) genes is the most economic and efficient method for controlling the disease. *Xa4* is a dominant *R* gene that provides durable resistance to bacterial blight. Cultivars with *Xa4* conferred resistance to almost all Chinese patho-types of *Xoo* except for patho-type C5 (Zhang [2009]). *Xa21* is another dominant bacterial blight gene, which was originally discovered in wild rice specie *Oryza longistaminata* (Ikeda et al. [1990]). IRBB21, an *Xa21* line in IR24 genetic background, showed resistance to all the known races of *Xoo* collected from India and the Philippines (Ikeda et al. [1990]). Both *Xa4* and *Xa21* genes are widely exploited *R* genes in Asian rice breeding programs for bacterial blight resistance (Datta et al. [2002]; Huang et al. [1997]; Luo et al. [2012]; Luo and Yin [2013]; Singh et al. [2001]; Suh et al. [2013]; Zhang et al. [2006]).

Although modern rice varieties have been planted worldwide, elite local rice varieties are still popular in many rice growing countries due to their good grain quality and adaptation to the local climate and environment. Cultivar Siputeh is a local rice variety still planted in Java and Sumatra islands of Indonesia. Siputeh produces long grain rice with good cooking and eating quality. However, the variety has low yield with tall plant type and long growth duration and is highly susceptible to biotic and abiotic stress (Suhartini [1991]). Here we report the genetic improvement of Siputeh for semi-dwarf phenotype, good grain quality and disease resistance to bacterial blight through marker-assisted breeding.

## Results

### Breeding of TS4

**Figure 1:**
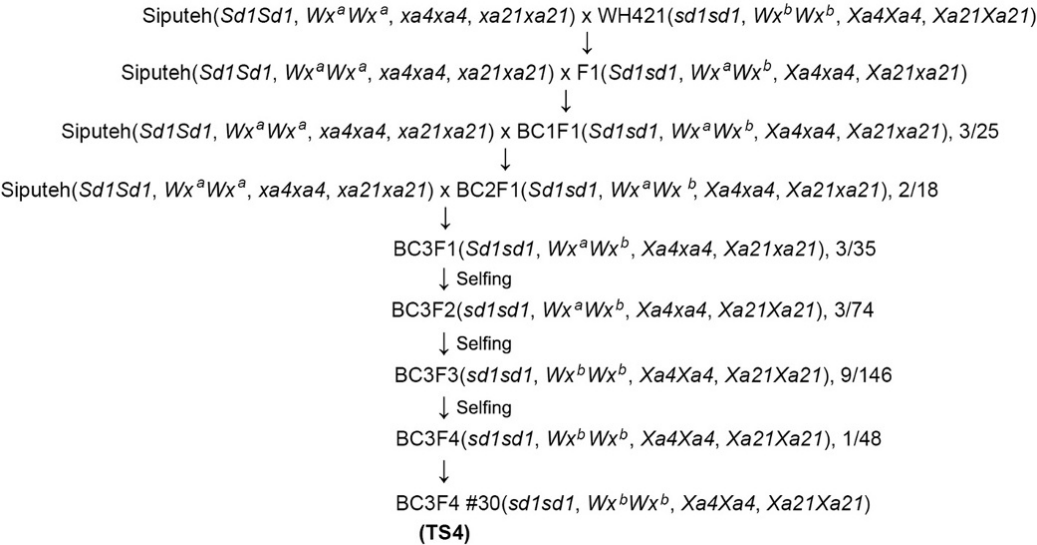
**Marker-assisted breeding of TS4.** The number of positive plants over the number of total plants screened for presence of molecular makers are indicated in the generation undergone marker-assisted selection. *Sd1* and *sd1*, the dominant and recessive alleles of the *Sd1* gene; *Wx*^a^ and *Wx*^b^, the alleles *a* and *b* of the rice *Wx* gene; *Xa4* and *xa4*, dominant and recessive alleles of the *Xa4* gene, respectively; *Xa21* and *xa21*, dominant and recessive alleles of the *Xa21* gene, respectively.

**Figure 2:**
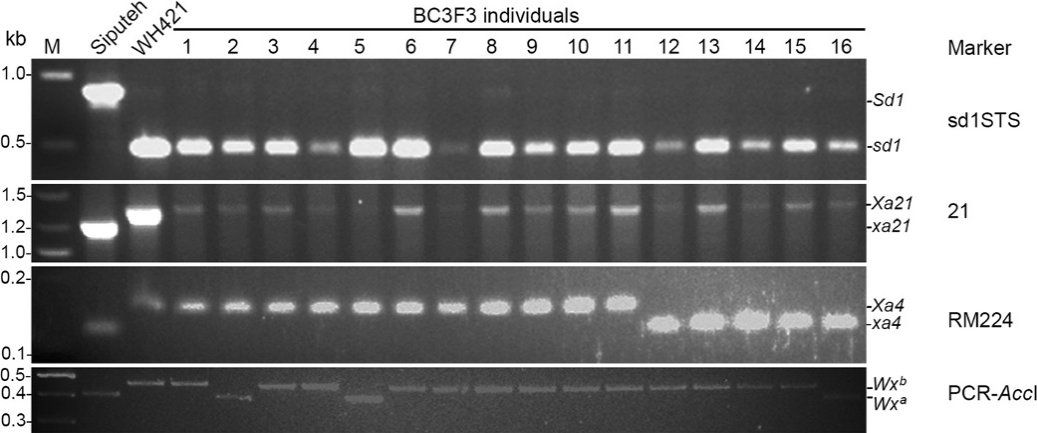
**Detection of molecular markers at the** ***sd1*** **,** ***Xa4, Xa21*** **and** ***Wx***^**b**^**loci in the B3F3 individuals.** The BC3F3 individuals were derived from the cross between Siputeh (recurrent female) and WH421 followed by backcrossing for 3 generations. The amplified PCR products were separated on a 1.5% w/v agarose gel for Marker sd1STS, a 2.0% agarose gel for Marker 21, and a 3.5% agarose gel for Marker PCR-*Acc* I and RM224.BC3F3 plants #1, 3, 4, 6–11 carry homozygous alleles at the *sd1*, *Xa4*, *Xa21* and *Wx*^b^ loci.

Marker-assisted breeding was conducted between cultivar Siputeh as the recurrent female and cultivar WH421 as the donor line for the *sd1, Wx*^b^*, Xa4* and *Xa21* genes (Figure 1). The genotypes at the *sd1, Wx, Xa4* and *Xa21* loci in each plant at different generations were determined with molecular markers as described in Table 1. In summary, 10 F1 plants, 3 BC1F1 plants, 2 BC2F1 plants and 3 BC3F1 were identified from F1, BC1F1, BC2F1 and BC3F1 generations, respectively (Figure 1). All these plants carried heterozygous alleles at the *sd1*, *Wx*^b^, *Xa4* and *Xa21* loci (Figure 1). One of the 3 BC3F1 plants (BC3F1 #6) was selected for self-pollination to generate a BC3F2 population, which consisted of 74 BC3F2 individuals. Three BC3F2 plants were identified from the BC3F2 population and they all carried homozygous alleles at the *sd1* and *Xa21* loci and heterozygous alleles at the *Wx*^b^ and *Xa4* loci (Figure 1). Plant BC3F2 #38 was selected to produce a BC3F3 population, which consisted of 146 BC3F3 individuals. Nine plants (BC3F3 #1, 3, 4, 6–11) were identified from the BC3F3 population and they all carried homozygous alleles at the *sd1, Wx*^b^*, Xa4* and *Xa21* loci (Figures 1 and 2). Plant BC3F3 #1 was selected to produce a BC3F4 population. The 48 individual plants in the BC3F4 population showed uniform morphological phenotype and similar growth duration. Plant BC3F4 #30 was designated as TS4 and selected for disease evaluation for resistance to bacterial blight and field trials (Figure 1).

**Table 1:** **Molecular markers used in this study**

Marker	Gene-of-interest	DNA sequence	Type of marker	Reference
sd1STS	*sd1*	F: 5′ACAAATACCCCACCCTCCTGCC3′	STS, co-dominant	(Luo and Yin [2013])
		R: 5′TAGTAGCCTCGCTCCACGCCC3′		
21	*Xa21*	F: 5′ATAGCAACTGATTGCTTGG3′	STS, co-dominant	(Chen et al. [2000])
		R:5′ GATCGGTATAACAGCAAAAC3′		
RM224^a^	*Xa4*	F:5′ ATCGATCGATCTTCACGAGG3′	SSR, co-dominant	(Sun et al. [2003])
		R:5′ TGCTATAAAAGGCATTCGGG3′		
PCR-*Acc* I^b^	*Wx*	F: 5′GCTTCACTTCTCTGCTTGTG3′	CAPS, co-dominant	(Cai et al. [2002])
		R: 5′ATGATTTAACGAGAGTTGAA3′		

F, forward primer; R, reverse primer; STS, sequence-tagged site; SSR, simple sequence repeat; CAPS, Cleaved amplified polymorphic sequence.

^a^The genetic distance between RM224 and the *Xa4* locus is 1.1 cM.

^b^CAPS marker at the *Wx* locus by digesting PCR product with *Acc* I to show polymorphism.

### Disease evaluation of TS4 for bacterial blight resistance

Twenty-eight *Xoo* strains were used to inoculate WH421, Siputeh and TS4. Siputeh was susceptible to all *Xoo* strains tested while WH421 conferred resistance or moderate resistance to 22 strains and was susceptible or moderate susceptible to 1947, GD1358, A3857, JW89011, K202 and PXO99 (Table 2). TS4 provided resistance or moderate resistance to 25 of the 28 *Xoo* strains tested and was only susceptible to A3857, JW89011 and K202 (Table 2). Compared to WH421, TS4 also provided enhanced resistance with shorter disease lesions to 1947, GD1358, A3857, JW89011, K202 and PXO99 (Table 2). It should be noted that neither WH421 nor TS4 could provide complete resistant to PXO99 (Table 2). The result was not consistent with the previous finding that the *Xa21* gene in either IR24 or TP309 genetic background conferred complete resistance to PXO99 (Gu et al. [2004]; Ikeda et al. [1990]; Song et al. [1995]; Wang et al. [1996]).

**Table 2:** **Disease evaluation of WH421, Siputeh and TS4 for resistance to** ***Xoo*** **strains**

		Lesion length (cm) and disease phenotype^a^		
Stains	Origin	WH421	Siputeh	TS4
1947	Africa	14.8 ± 7.4 (S)	24.1 ± 6.9 (S)	4.4 ± 4.5 (MR)
Aust-2031	Australia	2.8 ± 1.3 (R)	25.5 ± 8.4 (S)	1.3 ± 0.5 (R)
Aust-R3	Australia	1.8 ± 1.1 (R)	39.5 ± 8.8 (S)	1.0 ± 0.5 (R)
GD1358	China	7.0 ± 1.7 (MS)	23.9 ± 4.7 (S)	4.8 ± 3.6 (MR)
HB17	China	0.5 ± 0.3 (R)	31.2 ± 3.8 (S)	0.9 ± 0.6 (R)
HB21	China	1.7 ± 0.9 (R)	39.6 ± 6.7 (S)	1.3 ± 0.4 (R)
HLJ72	China	1.5 ± 0.6 (R)	16.2 ± 3.3 (S)	0.7 ± 0.4 (R)
JS49-6	China	0.9 ± 0.5 (R)	13.6 ± 4.9 (S)	1.3 ± 0.6 (R)
LN57	China	0.7 ± 0.4 (R)	30.7 ± 8.5 (S)	1.1 ± 0.8 (R)
NX42	China	1.3 ± 0.8 (R)	29.7 ± 5.6 (S)	1.5 ± 1.0 (R)
ZHE173	China	0.2 ± 0.1 (R)	34.2 ± 7.8 (S)	1.1 ± 0.8 (R)
CIAT1185	Columbia	3.5 ± 2.0 (MR)	10.0 ± 3.5 (S)	2.3 ± 0.7 (R)
A3842	India	4.3 ± 1.2 (MR)	37.9 ± 13.2 (S)	3.0 ± 1.1 (R)
A3857	India	18.8 ± 4.9 (S)	38.3 ± 7.4 (S)	10.5 ± 2.4 (S)
IXO56	Indonesia	6.0 ± 2.2 (MR)	30.3 ± 6.7 (S)	3.7 ± 2.0 (MR)
H75373	Japan	0.6 ± 0.6 (R)	25.5 ± 4.9 (S)	0.4 ± 0.1 (R)
T7174	Japan	0.6 ± 0.6 (R)	29.9 ± 6.7 (S)	0.6 ± 0.5 (R)
JW89011	Korea	16.1 ± 8.9 (S)	35.9 ± 7.0 (S)	9.2 ± 4.3 (S)
NXO260	Nepal	3.8 ± 6.1 (MR)	22.1 ± 2.6 (S)	1.1 ± 1.0 (R)
K202	Korea	15.1 ± 3.7 (S)	44.9 ± 10.7 (S)	17.0 ± 4.3 (S)
PXO71	Philippines	0.4 ± 0.3 (R)	37.8 ± 14.6 (S)	1.1 ± 0.7 (R)
PXO79	Philippines	0.7 ± 0.5 (R)	20.4 ± 7.5 (S)	2.8 ± 1.5 (R)
PXO86	Philippines	1.9 ± 0.8 (R)	28.7 ± 9.6 (S)	2.8 ± 1.2 (R)
PXO99	Philippines	11.8 ± 2.8 (S)	25.0 ± 7.1 (S)	5.9 ± 3.0 (MR)
PXO112	Philippines	0.6 ± 0.9 (R)	10.8 ± 3.3 (S)	1.1 ± 2.3 (R)
PXO113	Philippines	0.6 ± 0.4 (R)	27.8 ± 7.8 (S)	0.9 ± 0.6 (R)
Thai R7	Thailand	1.3 ± 0.5 (R)	13.2 ± 6.0 (S)	0.9 ± 0.7 (R)
Thai R2	Thailand	2.8 ± 1.3 (R)	35.4 ± 5.6 (S)	2.2 ± 1.4 (R)

^a^R, resistant (Lesion length ≤3.0 cm); MR, moderately resistant ( 3.0 cm < Lesion length ≤6.0 cm); MS, moderately susceptible (6.0 cm < Lesion length ≤9.0 cm); S, susceptible (Lesion length >9.0 cm).

### Field evaluation of TS4 and Siputeh

**Figure 3:**
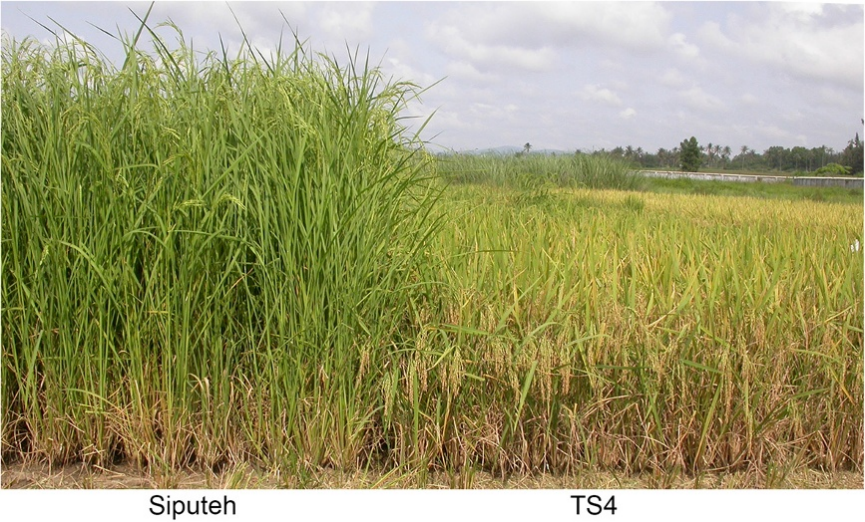
**Plant height and maturation time of Siputeh and TS4 in the field.** The semi-dwarf TS4 plants matured earlier than Siputeh with tall plant type. Picture was taken in the field in Lingshui, China, at 162 days after sowing.

The agronomic traits of TS4 and Siputeh were evaluated in two field trials and different growth seasons conducted in Banda Aceh, Indonesia and Lingshui, China, respectively. Due to presence of the *sd1* gene, TS4 (123.7 ± 2.1 cm) had shorter plant height than Siputeh (208.3 ± 3.9 cm) when they were grown in Banda Aceh (Table 3). The difference in plant height between Siputeh (214.6 ± 4.4 cm) and TS4 (106.7 ± 1.1 cm) became even greater when they were grown in Lingshui under short-day condition during the winter season (Figure 3). TS4 (117 days) had shorter growth duration than Siputeh (160 days) when they were planted in Banda Aceh, Indonesia (Table 3). However, both TS4 (162 days) and Siputeh (182 days) had long growth duration when they were planted in Lingshui under short-day condition during the winter (Table 3). TS4 produced more productive panicles per plant with higher seed-setting rate but fewer grain number per panicle than Siputeh grown in both field trials (Table 3). The greater number of productive panicles per plant and higher seed-setting rate also collectively contributed to the higher grain yield of TS4 than that of Siputeh grown in both field trails (Table 3). TS4 (27.6 g) had similar 1000-grain weight to Siputeh (27.4 g) when they were grown in Banda Aceh (Table 3). However, the 1000-grain weight of TS4 (34.1 g) were slightly heavier than that of Siputeh (31.7 g) when they were grown in Lingshui (Table 3). In summary, TS4 showed better agronomic traits with shorter plant height and growth duration and higher yield than Siputeh.

**Table 3:** **Agronomic traits of Siputeh and TS4 plants grown in field trials**

	Field trial 1^a^			Field trial 2^b^		
Agronomic trait	Siputeh	TS4	***t***-test^c^	Siputeh	TS4	***t***-test
Growth duration (days)	160	117	-	182	162	-
Plant height (cm)	208.3 ± 3.9	123.7 ± 2.1**	33.081	214.6 ± 4.4	106.7 ± 1.1**	41.120
Productive panicles per plant	6.5 ± 0.8	9.0 ± 0.4**	4.841	4.8 ± 0.2	5.7 ± 0.5*	3.298
Grain number per panicle	137.3 ± 3.4	129.7 ± 2.9*	2.946	172.4 ± 11.5	139.0 ± 16.3*	2.902
Seed-setting rate (%)	70.1 ± 2.8	80.6 ± 2.6**	4.760	70.5 ± 1.2	82.8 ± 2.0**	9.097
1000-grain weight (g)	27.4 ± 0.7	27.6 ± 0.4	0.430	31.7 ± 0.6	34.1 ± 0.5**	8.978
Yield (t/ha)	4.3 ± 0.3	6.5 ± 0.1**	12.050	6.6 ± 0.7	8.0 ± 0.2*	3.538

^a^Field trial 1 was conducted in Banda Aceh, Indonesia, in the wet season of 2011/2012 (November 2011 to April 2012).

^b^Field trial 2 was conducted in Lingshui, China, in the winter season of 2013/2014 (November 2013 to May 2013).

^c^*t*-test is the comparision between Siputeh and TS4. T_0.05, 4_ = 2.776, T_0.01, 4_ = 4.604. “**” and “*” stand for significance difference at 0.01 and 0.05 probability levels, respectively.

### Comparison of grain quality between TS4 and Siputeh

The grain quality of TS4 and Siputeh was measured using rice grains harvested from the two field trials. Both TS4 and Siputeh produce long grain rice with similar grain length and length-to-width ratio (Table 4). TS4 also has significantly lower degree of chalkiness than Siputeh in both field trials (Table 4). TS4 has lower amylose content (15.3-17.8%) than Siputeh (26.3-28.9%), due to the presence of the *Wx*^b^ gene in TS4 (Table 4). For the rice grains harvested in field trial in Banda Aceh, TS4 has soft gel consistency while Siputeh had medium gel consistency (Table 4). Both TS4 and Siputeh had intermediate gelatinization temperature even though TS4 had slightly lower akali spreading value than Siputeh (Table 4). The results collectively showed that TS4 has better grain quality than Siputeh in terms of degree of chalkiness and amylose content.

**Table 4:** **Grain quality of Siputeh and TS4**

	Field trial 1^a^			Field trail 2^b^		
Traits	Siputeh	TS4	***t***-test^c^	Siputeh	TS4	***t***-test
Grain length (mm)^d^	7.4 ± 0.1 (Long)	7.6 ± 0.1 (Long)	2.449	7.2 ± 0.1 (Long)	7.3 ± 0.1 (Long)	1.225
Length-to-width ratio^e^	3.2 ± 0.1 (Slender)	3.1 ± 0.1 (Slender)	1.225	3.4 ± 0.1 (Slender)	3.2 ± 0.1 (Slender)	2.449
Degree of chalkiness (%)^f^	23.0 ± 2.0 (9)	12.0 ± 1.0** (5)	18.249	16.3 ± 0.6 (5)	9.3 ± 0.3** (1)	18.074
Amylose content (%)^g^	26.3 ± 0.1 (High)	15.3 ± 0.2** (Low)	67.361	28.9 ± 0.2 (High)	17.8 ± 0.2** (Low)	67.973
Gel consistency (mm)^h^	51.0 ± 1.6 (Medium)	90.0 ± 2.7** (Soft)	23.735	68.8 ± 5.0 (Soft)	94.5 ± 0.9** (Soft)	8.762
Alkali spreading value						
and gelatinization temperature^i^	5.0 ± 0.1 (Intermediate)	4.5 ± 0.1** (Intermediate)	8.660	5.0 ± 0.0 (Intermediate)	4.0 ± 0.1** (Intermediate)	6.124

^a^Field trial 1 was conducted in Banda Aceh, Indonesia, in the wet season of 2011/2012 (November 2011 to April 2012).

^b^Field trial 2 was conducted in Lingshui, China, in the winter season of 2013/2014 (November 2013 to May 2013).

^c^*t*-test is the comparision between Siputeh and TS4. T_0.05, 4_ = 2.776, T_0.01, 4_ = 4.604. “**” and “*” stand for significance difference at 0.01 and 0.05 probability levels, respectively.

^d^Category of grain length: Very long, grain length > 7.5 mm; Long, 6.6 mm < grain length ≤ 7.5 mm; Medium, 5.5 mm < grain length ≤ 6.6 mm; Short, grain length ≤ 5.5 mm.

^e^Grain shape based on length-to-width ratio: Slender, length-to-width ratio > 3.0; Medium, 2.0 < length-to-width ratio ≤ 3.0; Bold, length-to-width ratio ≤ 2.0.

^f^Scale for degree of chalkiness: 0, degree of chalkiness = 0; 1, 0 < degree of chalkiness ≤ 10%; 5, 10% < degree of chalkiness ≤ 20%, 9, degree of chalkiness > 20%.

^g^Classification of amylose content: Waxy, amylose content ≤ 2%; Very low, 2% < amylose content ≤ 9%; Low, 9% < amylose content ≤ 20%; Intermediate, 20% < amylose content ≤ 25%; High, amylose content > 25%.

^h^Classification of gel consistency: Soft, gel consistency > 60 mm; Medium, 40 mm < gel consistency ≤ 60 mm; Hard, gel consistency ≤ 40 mm.

^i^Grade of gelatinization temperature estimated by alkali spreading value: High (74.5°C ≤ gelatinization temperature < 80°C), 1 ≤ alkali spreading value < 2.5; Intermediate high (74°C ≤ gelatinization temperature < 74.5°C), 2.5 ≤ alkali spreading value < 3.5; Intermediate (70°C ≤ gelatinization temperature < 74°C), 3.5 ≤ alkali spreading value < 5.5; Low (gelatinization temperature < 70°C), 5.5 ≤ alkali spreading value ≤ 7.

## Discussion

Marker-assisted selection is a molecular breeding process whereby a molecular marker based on DNA variation is used for indirect selection of an interest agronomic trait. Marker-assisted selection is not affected by environments and can be conducted at any plant growth stage. It is especially useful for selection of recessive genes and biochemical traits in heterozygous plants. In this study, the *sd1* gene is a partially recessive gene that controls semi-dwarf phenotype. It is difficult or impractical to select the *sd1* gene in the backcrossing progeny according to plant height, especially under greenhouse condition. Similarly, the amylose content in rice grains can only be directly detected after harvesting, however, we need to know the genotype for crossing purpose before flowering. As for the pyramiding of *Xa4* and *Xa21* for bacterial blight resistance, both *R* genes have different resistance specificities and their resistance spectrums are over-lapped. It would be impractical to select the two *R* genes in a single line by conducting disease evaluation and phenotypic selection. To overcome these difficulties, we chose marker-assisted selection approach, which enable us to perform precisely and effectively multiple gene selection and/or pyramiding in limited breeding time.

Compared with Siputeh, the significant change in TS4 is the semi-dwarf plant type, which is mainly resulted from the introduction of the *sd1* gene. The semid-warf plant type of rice indiredtly contributes to lodging resistance in the field. It also increases grain-straw ratio, which ultimately results in higher yield. In the present study, TS4 produced higher yields than Siputeh in both field trials conducted in different location (Table 3). The results are consistent with the results of previously studies on the *sd1* gene introduced in other varieties (Hedden [2003]; Luo and Yin [2013]; Spielmeyer et al. [2002]). TS4 also showed significant reduction in growth duration than Siputeh. The growth duration of TS4 was about four months when it was grown in Banda Aceh, Indonesia (Table 3), which was similar to most of the typical modern rice varieties grown in tropical region. Short-growth duration makes it easier for farmers to plan growth season, synchronize sowing and harvest time with other varieties and manage diseases and insects in the field.

Like the *Xa4* and *Xa21* donor line WH421, TS4 provided broad-spectrum resistance to multiple *Xoo* strains tested in this study. It was noted that WH421 were susceptible or moderate susceptible to 1947, PXO99 and GD1358 while TS4 provided moderate resistance to the three *Xoo* strains (Table 2). It seems that either *Xa4* or *Xa21* or both *R* genes performed better for resistance to *Xoo* in TS4 than that in WH421. Previously, it was found that the *Xa21* gene in IRBB21 conferred complete resistance to PXO99 (Gu et al. [2004]; Ikeda et al. [1990]; Song et al. [1995]), however, in this study, neither WH421 nor TS4 could confer full resistance to the *Xoo* strain. It has been reported that the *R* gene-mediated resistance to *Xoo* in rice may be influenced by rice genetic backgrounds and developmental stages (Cao et al. [2007]). For instance, the *Xa21*-containing rice line Minghui was still susceptible to PXO99 (Zhang et al. [2006]).

The introduction of the *sd1* gene to Siputeh would significantly reduce its plant height and growth duration, which might affect rice grain quality. To overcome this potential shortcoming, we also introduced the *Wx*^b^ gene into TS4 through marker-assisted selection. Indeed, the *Wx*^b^ gene significantly reduced the amylose content in TS4. Amylose content strongly affects the cooking and eating quality of rice. Rice with amylose content at 25-30% usually tends to be firm and dry after cooking, rice with amylose content at 20-25% tends to be softer and stickier and rice with amylose content at <20% is generally quite soft and sticky. TS4 has amylose content at 15.3-17.8% and shows soft gel consistency. The results indicate that TS4 is generally softer and stickier than Siputeh, which has amylose content at 26.3-28.9% and shows medium to soft gel consistency.

## Conclusion

An improved rice line, designed as TS4, has been developed to contain semi-dwarf gene *sd1*, low amylase content gene *Wx*^b^ and bacterial light resistance genes *Xa4* and *Xa21* through marker-assisted selection. TS4 has semi-dwarf phenotype with reduced growth duration, produces high yield with good grain quality and provides broad-spectrum resistance to *Xoo* strains. The development of TS4 enriches the diversity of local rice varieties with high yield potential and good grain quality.

## Methods

### Plant materials and growth condition

Rice variety Siputeh is a local cultivar collected in Aceh province, Indonesia. Rice variety WH421 carrying *sd1*, *Wx*^b^, *Xa4* and *Xa21* genes is a paternal line of hybrid rice in Mianhui 725 backgroud (Luo et al. [2012]). Rice plants were grown in the greenhouse at 32°C for 12.5 h (light) and 25°C for 11.5 h (dark).

### PCR-based molecular markers

The molecular markers used in this study include the codominant STS (Sequence-Tagged Sites) marker sd1STS for the *sd1* gene (Luo and Yin [2013]), the co-dominant microsatellite DNA marker RM224 for the *Xa4* gene (Sun et al. [2003]), the co-dominant STS marker 21 for the *Xa21* gene (Chen et al. [2000]) and the co-dominant CAPS (Cleaved Amplified Polymorphic Sequences) marker PCR-*Acc* I for the *Wx*^a^ and *Wx*^b^ genes (Cai et al. [2002]). Oligo primers for PCR amplification of the molecular markers are listed in Table 1.

### PCR amplification

PCR was performed on a PTC-100 programmable thermal controller (MJ Research). The PCR reaction mixture of 20 μl consisted of 1–100 ng of rice genomic DNA, 0.2 mM each of dNTPs, 0.2 μM of each primer, 2 μl of 10 × PCR buffer [500 mM KCl, 100 mM Tris–HCl (pH 8.3), 20 mM MgCl_2_] and 0.5 unit of Taq polymerase. For PCR amplification of markers sd1STS, 4 ul of 5 × Q-solution (QIAGEN) was added to the PCR reaction mixture. Template DNA was initially denatured at 94°C for 2 min followed by 35 cycles of PCR amplification with the following parameters: a 30 s of denaturation at 94°C, a 40 s of primer annealing at 55°C for markers 21, RM224 and PCR-*Acc* I, 65°C for maker sd1STS, and 1 min of primer extension at 72°C for markers RM224 and PCR-*Acc* I, and 1.5 min for markers 21 and sd1STS. Finally, the reaction mixture was maintained at 72°C for 5 min of primer extension before completion. The PCR product of the marker PCR-*Acc* I was digested by *Acc* I for 4 h. The amplified products were electrophoretically resolved on a 1.5% agarose gel for marker sd1STS, a 2.0% agarose gel for marker 21, and a 3.5% agarose gel for marker PCR-*Acc* I and RM224 in 1 × TAE buffer.

### Bacterial inoculation and disease scoring

*Xoo* strains were grown on PSA medium (10 g/l peptone, 10 g/l sucrose, 1 g/lglutamic acid, 16 g/l bacto-agar, and pH7.0) for about 60 hours at 28°C. Bacterial cells were suspended in sterile water and diluted to an opticla density (OD) at 600 nm of 0.5. Plants were inoculated by the leaf-clipping method (Kauffman et al. [1973]). Lesion length (L.L.) was measured at 14 days after inoculation. The disease symptoms were scored as resistant (R, L.L. ≤ 3.0 cm), moderately resistant (MR, 3.0 cm < L.L. ≤ 6.0 cm), moderately susceptible (MS, 6.0 cm < L.L. ≤ 9.0 cm) or susceptible (S, L.L. > 9 cm).

### Field trial design and collection of important agronomic traits

Field trials were conducted in Banda Aceh, Indonesia in the season of 2011/2012 (November 2011 - March 2012) and in Lingshui, China in the winter season of 2013/2014 (November 2013 - May 2014), respectively. Rice lines were tested in replicated plots in each field trial. The plot size was 4.2 m × 3.8 m and the plant spacing was 20 cm × 20 cm in Banda Aceh, while the plot size was 4 m × 4 m and the plant spacing was 16.7 cm × 16.7 cm in Lingshui. Thirty plants (3 × 10 plants/per plot) were randomly selected for each tested line and scored for important agronomic traits, including growth duration, plant height, productive panicle per plant, number of grains per panicle, seed setting rate, weight of 1000 grains and grain yield per hectare. Statistical analysis was performed using a two-tailed *t-* test for independent samples.

### Evaluation of grain quality

Rice grain quality parameters, including grain length, the ratio of length to width (L/W), chalkiness degree, amylose content (AC), gel consistency (GC) and alkali spreading value (ASV) were measured by the methods described previously (Cruz and Khush [2000]).

## Authors’ information

YL is the Senior Research Officer of Temasek Life Science Laboratory (TLL) and the Professor of Anhui Rice Research Institute (ARRI), Anhui Academy of Agricultural Sciences (AAAS), with over 25 years of experiences on rice breeding; SZ is the Professor of Agriculture Faculty, Syiah Kuala University (SKU); BB is the Associate Professor of Agriculture Faculty, SKU; TM is the Associate Professor of ARRI, AAAS; ZL is the Director and Professor of ARRI, AAAS; JY is the Professor of ARRI and the President of AAAS; ZY is the Senior Principal Investigator of TLL.

## Authors’ original submitted files for images

Below are the links to the authors’ original submitted files for images. Authors’ original file for figure 1 Authors’ original file for figure 2 Authors’ original file for figure 3

## Abbreviations

CAPS: Cleaved amplified polymorphic sequence
DNA: Deoxyribonucleic acid
GBSSI: Granule-bound starch synthase I
MR: Moderately resistant
MS: Moderately susceptible
OD: Opticla density
PCR: Polymerase Chain Reaction
R: Resistant
S: Susceptible
SSR: Simple sequence repeat
STS: Sequence-tagged site
*Xoo*: *Xanthomonas oryzae* pv. *oryzae*
