# Supplementary figures and images for: Marker-assisted breeding of Indonesia local rice variety Siputeh for semi-dwarf phonetype, good grain quality and disease resistance to bacterial blight
Source: Rice (N Y). 2014 Dec 18;7:33. doi: 10.1186/s12284-014-0033-2 (PMC4884010; doi:10.1186/s12284-014-0033-2)

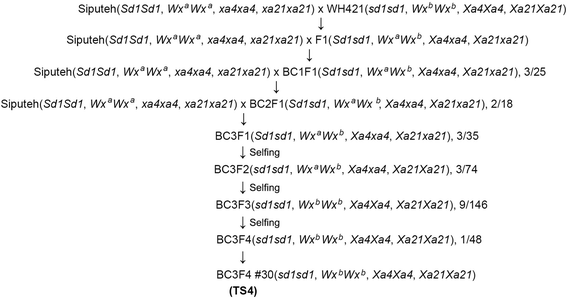

Supplement: Supplementary file 1 — Authors’ original file for figure 1 [file 12284_2014_33_MOESM1_ESM.gif]

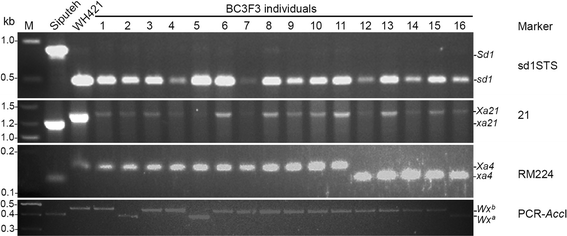

Supplement: Supplementary file 2 — Authors’ original file for figure 2 [file 12284_2014_33_MOESM2_ESM.gif]

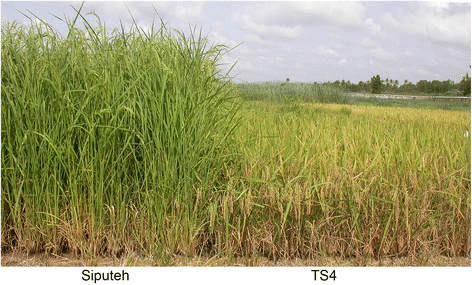

Supplement: Supplementary file 3 — Authors’ original file for figure 3 [file 12284_2014_33_MOESM3_ESM.gif]
